# Adult-Onset Syringomyelia in Dandy-Walker Malformation Patients: Clinical Insights and Literature Review

**DOI:** 10.3390/brainsci15050456

**Published:** 2025-04-26

**Authors:** Bojana Zivkovic, Mirko Micovic, Marko Todorovic, Jelena Kostic, Vladimir Bascarevic

**Affiliations:** 1 Clinic of Neurosurgery, University Clinical Center of Serbia, Dr Koste Todorovica Street 4, 11000 Belgrade, Serbia; 2 Faculty of Medicine, University of Belgrade, Dr Subbotica Street 8, 11000 Belgrade, Serbia

**Keywords:** intracranial cyst, fenestration, posterior fossa malformation, syrinx

## Abstract

**Introduction:** The presence of syringomyelia associated with Dandy-Walker malformation is rarely described in adults. **Case report:** We report a case of a 28-year-old woman with a history of Dandy-Walker malformation who developed syringomyelia. She had been previously treated in childhood with a ventriculoperitoneal and cystoperitoneal shunt for hydrocephalus, but over time she developed progressive neurological symptoms, including numbness and weakness in the upper extremities. Magnetic resonance imaging revealed a syrinx extending from C4 to T1 associated with large posterior fossa cyst. The patient was treated with cyst fenestration and cystoperitoneal shunts were removed, with complete resolution of symptoms and disappearance of syrinx. **Discussion:** A literature review revealed only 6 cases of syringomyelia associated with Dandy-Walker malformation in adults. The pathophysiology of this entity is multifactorial and may be related to obstructed cerebrospinal fluid flow, altered pressure dynamics, and formation of arachnoid adhesions. **Conclusions:** Individualized surgical approaches are essential for optimizing outcomes in this rare condition. Further research is needed to standardize treatment protocols and clarify underlying mechanisms and help to improve the management of these patients.

## 1. Introduction

Dandy-Walker malformation (DWM) is a rare congenital anomaly of the posterior fossa, affecting approximately 1 in 25,000 to 30,000 live births [1]. DWM is characterized by cerebellar vermis dysgenesis, a posterior fossa cyst, and often hydrocephalus, which can result in elevated intracranial pressure. Although DWM is typically diagnosed in early childhood, some cases may remain undetected until adulthood [2]. The association between DWM and syringomyelia is rare, with only a few cases reported in the literature, especially involving adults [3].

In adult patients, the clinical presentation of DWM-associated syringomyelia can be highly variable, ranging from asymptomatic and incidental findings to severe neurological deficits [4]. Common symptoms include headache, sensory-motor disturbances in the upper extremities, and gait abnormalities [3,5]. Diagnosis is often delayed due to the insidious onset of symptoms and the rarity of this condition. The management of DWM-associated syringomyelia in adults remains challenging, with no standardized treatment protocol established [6]. Surgical approaches vary widely, including posterior fossa decompression, syrinx shunting, and cerebrospinal fluid diversion procedures such as ventriculoperitoneal and cystoperitoneal shunts [3,7]. The choice of surgical intervention depends on the specific anatomical features, clinical presentation, and the surgeon’s experience.

We present a rare manifestation of acquired syringomyelia in an adult woman who had previously been diagnosed with and surgically managed for Dandy-Walker malformation. This case is particularly noteworthy because the patient has been under our hospital’s care since early childhood, giving us unique insight into the development of syringomyelia. Alongside the case presentation, this paper aims to review the current literature on DWM-associated syringomyelia in adults, focusing on pathogenesis, clinical features, and therapeutic strategies. By analyzing the available evidence, we seek to enhance our understanding of this uncommon condition and provide insights that may guide future research and clinical management.

## 2. Case Report

A 28-year-old woman presented in May 2018 with a 6-month history of progressive bilateral upper extremity numbness and intermittent arm weakness, which contributed to her clumsiness. She also described posterior cervical discomfort characterized by a stretching sensation. At the age of 2, the patient underwent a ventriculoperitoneal (VP) shunt procedure to treat hydrocephalus related to a Dandy-Walker malformation. Subsequently, at the age of 9, the patient began to experience progressively worsening occipital headaches, episodes of nausea, and unsteady gait. MR showed an enlarging posterior fossa cyst, prompting the implantation of a cystoperitoneal (CP) shunt. Over the years, she underwent three additional surgical interventions at the same medical institution for shunt revisions due to proximal catheter obstruction, with the most recent intervention occurring four years prior. Following these procedures, the patient remained stable, requiring no further shunt revisions, and was monitored annually, reporting only occasional mild numbness in the upper limbs.

**Figure 1:**
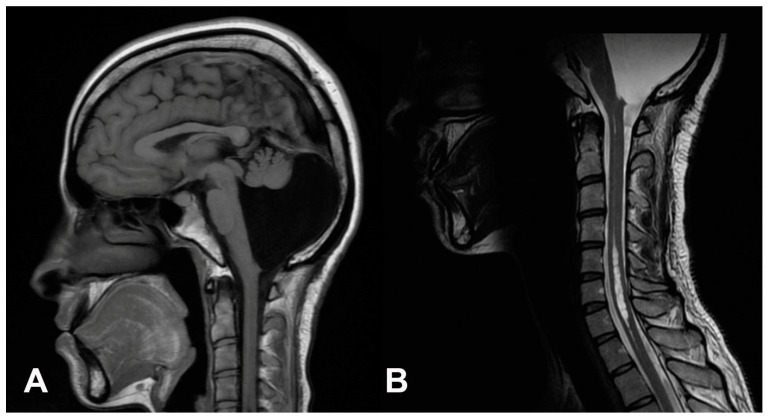
Preoperative MRI. (**A**): Brain MRI showing normal ventricle size without hydrocephalus. The cyst occupies nearly the entire posterior fossa, alongside hypoplastic cerebellar vermis; (**B**): cervical spine MRI showing syrinx extending from C4 to T1 vertebral levels.

Detailed examination revealed mild bilateral optic nerve atrophy with right-predominant horizontal nystagmus on lateral gaze. She also had hypoesthesia on the right C8 dermatome. The other clinical findings were unremarkable. Imaging studies revealed syringomyelia along the cervicothoracic spinal cord and a membranous structure around the craniocervical junction. The head MRI showed the correct position of the ventricular catheter in the right lateral ventricle. However, the posterior fossa was almost completely occupied by the cyst, and the hypoplastic cerebellar vermis and hemispheres were pushed forward and downward with syrinx extending from C4 to T1 level (Figure 1).

**Figure 2:**
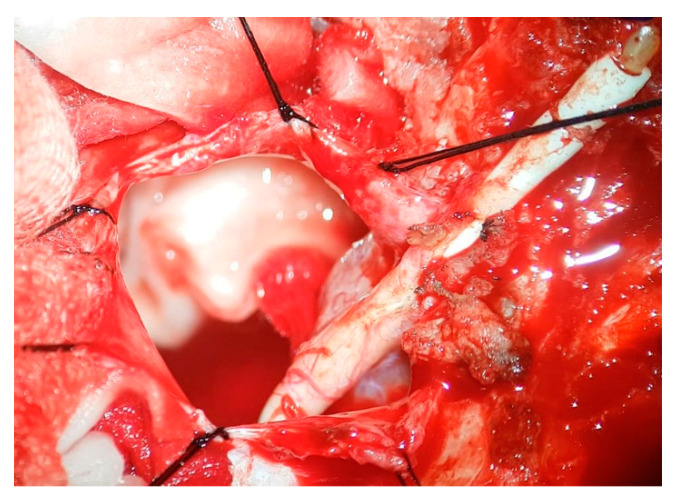
Intraoperative image. Complete occlusion of the proximal catheter by fibrous tissue.

The patient was admitted for surgery, with the treatment plan involving the removal of the cystoperitoneal shunt and an exploration of the wall and content of the DW cyst. Intraoperatively, the proximal catheter was found to be completely occluded (Figure 2), with numerous scattered thick adhesions in the posterior fossa. The thick membrane toward the cisterna magna was opened bilaterally and fenestrated, and the aqueduct opening was explored and also found to be completely obliterated (Video S1). The posterior fossa shunt was removed at the end of the surgical procedure.

**Figure 3:**
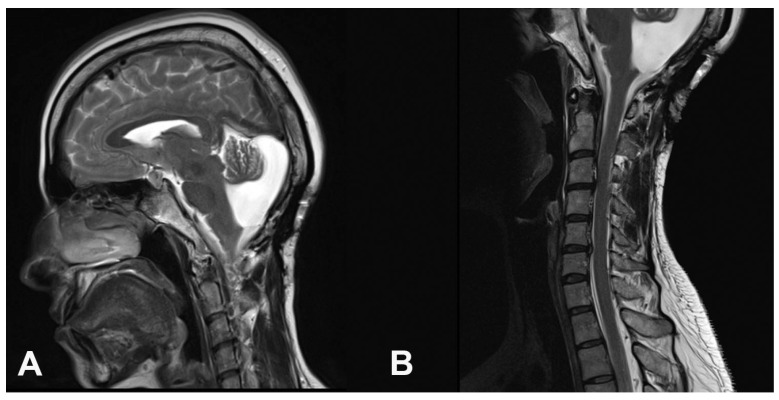
Follow-up MRI six months after the surgery showed a decrease in the size of the posterior fossa cyst (**A**) and complete resolution of the syrinx (**B**).

The postoperative period was uneventful, and all symptoms resolved completely within a few weeks. Follow-up MRI was performed six months after the surgery and showed a considerable reduction in the size of the posterior fossa cyst and complete resolution of the syrinx (Figure 3). The patient remained asymptomatic throughout a six-year follow-up period.

## 3. Literature Review

The association between DWM and syringomyelia has been reported in 26 individuals, and most of them occurred in infants or adolescents [8,9,10,11,12,13,14,15]. Only six adult cases have been documented in the existing literature, excluding autopsy findings and pediatric patients (Table 1).

Gardner et al. [11] described a 24-year-old female with progressive gait instability, bulbar symptoms, and dissociated sensory loss. Surgical exploration revealed a diverticulum of the fourth ventricle caused by obstruction at the foramen of Magendie and a compressive fibrous band at the foramen magnum. The patient made a good recovery, and the sensory loss had been reduced, but she still had some degree of ataxia.

Baker et al. [8] reported a 25-year-old female with cervicothoracic pain and lower limb weakness who underwent surgery for decompression of a Dandy-Walker cyst and plugging of the obex. Postoperative follow-up showed marked symptomatic improvement.

Hammond et al. [12] presented a 39-year-old male with occipital headaches and a history of DWM treated in infancy. Imaging revealed shunt disconnection and a large syrinx. Revision of the cystoperitoneal shunt resulted in the resolution of symptoms and a reduction in syrinx size.

Owler et al. [13] documented a 29-year-old female with headache and bilateral upper limb paresthesias. MRI demonstrated a posterior fossa cyst causing cervicomedullary compression and syringomyelia. Decompression and cyst fenestration resulted in complete resolution of both clinical symptoms and the syrinx on imaging.

Zhang et al. [15] described a 33-year-old male with progressive gait disturbance, cognitive impairment, and urinary incontinence. Imaging showed ventriculomegaly, a DWM cyst, and syringomyelia. A two-stage surgical approach, posterior fossa arachnoid adhesiolysis followed by endoscopic third ventriculostomy, led to complete clinical resolution and significant reduction of the syrinx.

Wang et al. [14] reported a 19-year-old male with upper limb weakness and tremor. MRI revealed cystic dilation of the 4th ventricle, cerebellar vermis hypoplasia, and syringomyelia. Surgical decompression, arachnoid adhesiolysis, and spinal cordostomy led to rapid neurological improvement and radiographic resolution.

## 4. Discussion

The co-existence of syringomyelia and DWM represents a challenge both for treatment and prognosis and prompts interesting questions about the pathogenesis and the best surgical approach. While the association between DWM and syringomyelia is rare, it may be underdiagnosed due to the subtlety of symptoms and the complexity of the condition [16]. MRI is the most utilized imaging modality, and is further supplemented by cine MRI [17]. Advances in imaging techniques have improved the ability to diagnose these conditions early, allowing for timely intervention and better outcomes.

### 4.1. Syrinx Formation Mechanisms in Dandy-Walker Malformation

Hydromyelia and syringomyelia are sometimes used interchangeably. On imaging, the two conditions may appear similar; however, from a pathophysiological and management standpoint, the distinction is important. Hydromyelia refers to a dilatation of the central canal of the spinal cord, typically lined by ependymal cells. It is usually considered a developmental or congenital anomaly and may be asymptomatic. The cavity in hydromyelia is in continuity with the fourth ventricle and lies strictly within the anatomical boundaries of the central canal. In contrast, syringomyelia refers to the presence of a fluid-filled cavity within the spinal cord parenchyma, which is not lined by ependymal cells and may or may not communicate with the central canal. Syringomyelia is typically associated with conditions such as Chiari malformation, arachnoiditis, trauma, or—as in our case—posterior fossa malformations like Dandy-Walker. Syringomyelia is always the consequence of a primary disorder, causing progressive neurological deficits if untreated [15]. The co-occurrence of syringomyelia and DWM presents unique diagnostic and therapeutic challenges, due to poorly understood underlying pathophysiological mechanisms [18]. Since Gardner’s first report in 1957, only 26 cases of DWM-associated syringomyelia have been described in the literature, with the majority derived from autopsy series and pre-MRI era studies [3,19]. The advent of magnetic resonance imaging (MRI) has significantly improved our ability to detect and characterize this rare association, shedding light on potential pathogenic mechanisms and guiding surgical interventions [20].

A comprehensive theory that sufficiently clarifies various scenarios leading to syrinx formation in DWM remains elusive. The syrinx formation mechanisms are diverse and encompass various theories that elucidate the underlying processes. The intramedullary pulse pressure theory, proposed by Greitz [17], serves as a foundational explanation for syrinx formation. This theory suggests that the obstruction of cerebrospinal fluid flow within the spinal subarachnoid space, often due to conditions like fibrosis or arachnoiditis, leads to the development of syrinxes. In cases of complete obstruction, the CSF pressure pulse transmits to the spinal cord, causing widening of the parenchyma due to increased pressure from centrifugal forces. Partial obstruction allows for some CSF flow, leading to increased flow speed and decreased pressure in the narrowed channel (Bernoulli’s principle) and creating a suction effect (Venturi effect). Over time, the accumulation of fluid will lead to the dilation of the central canal and disrupt capillary circulation, enlarge the Virchow–Robin spaces, and decrease CSF absorption, ultimately resulting in the formation of a syrinx.

The obstruction of cerebrospinal fluid flow in DWM at the level of the fourth ventricle can disrupt normal CSF circulation and lead to uncontrolled hydrocephalus with increased intracranial pressure, which is a critical factor in the development of syringomyelia, and can disrupt normal CSF circulation, leading to the formation of a syrinx within the spinal cord [21]. In this scenario described by Milhorat et al. [4] increased intracranial pressure causes the central canal to expand, forming what is referred to as a “fifth ventricle”. This mechanism is particularly relevant in cases where there is an obstruction distal to the fourth ventricle, further exacerbating pressure gradients. The obstruction of the foramen magnum, often due to the herniation of the posterior fossa cyst, is also a significant factor in syrinx formation. This obstruction can block normal CSF flow, leading to syringomyelia [12,22]. The lower part of the cyst associated with DWM herniates downward, potentially obstructing CSF flow and leading to syrinx formation [10].

Cystoperitoneal and ventriculoperitoneal shunts are common treatments for hydrocephalus in DWM. However, these procedures can sometimes exacerbate syrinx formation if they do not adequately address the underlying cerebrospinal fluid flow obstruction [9,22].

Lee et al. [23] proposed arachnoid adhesions as an additional contributing factor to syrinx formation in DWM. An arachnoid web formed at the cervicomedullary junction, likely due to a previous intraventricular hemorrhage, may obstruct normal CSF flow and tether the brainstem. These adhesions impair CSF pressure gradients and promote fluid accumulation within the spinal cord, leading to syrinx formation. This mechanism was confirmed using phase-contrast cine MRI, which demonstrated disturbed CSF flow around the web.

Levine advocated for the concept of the spinal subarachnoid space and the neuroaxis venous system, which comprises two fluid columns in hydrostatic equilibrium [24]. In cases of foramen magnum obstructions, the “transmural pressure displacement” leads to mechanical stress on the venous system, causing disruption to the blood–spinal cord barrier and resulting in leakage of an ultrafiltrate of blood, which contributes to syrinx formation.

Vascular changes may also contribute to syringomyelia in DWM. Reduced craniospinal compliance, altered venous drainage, and increased resistance in the perivenous glymphatic outflow pathway may lead to resistance to the fluid’s movement and contribute to the formation of the syrinx [25].

Regarding our particular case, it seems most likely that the combination of the above-mentioned factors led to syrinx formation. The initial disturbance in cerebrospinal fluid (CSF) dynamics was likely due to inadequate shunt function, given the patient’s history of multiple shunt revision surgeries for insufficiency. However, the delayed onset of syringomyelia despite prolonged periods of shunt dysfunction suggests the involvement of an additional contributing factor. Intraoperative findings revealed extensive arachnoid scarring and a dense membranous obstruction at the level of the posterior fossa, impeding CSF communication with the spinal subarachnoid space. While the precise mechanism remains uncertain, this thickened membrane may represent a secondary factor that impeded CSF flow and predisposed the patient to syrinx formation.

### 4.2. Clinical Features

The clinical presentation of DWM associated with syringomyelia varies significantly among patients. In infants, common symptoms include headache, vomiting, cranial nerve deficit, hemiparesis, macrocrania, delayed motor milestones, and, in some cases, fecal incontinence [14,15]. In adults, however, clinical manifestations are more diverse, and patients may experience unsteady gait and ataxia, cognitive impairments with memory decline, sensory deficits including pain, numbness, weakness or tremor in the hands, urinary incontinence, and ocular symptoms such as visual field defects [12,13,14,15,26,27]. Our patient had mild bilateral optic atrophy and right-predominant horizontal nystagmus, which could most likely be explained by chronic intracranial hypertension affecting the visual pathways, possibly exacerbated during periods of intermittent shunt dysfunction. The nystagmus could be explained by a dysfunction of the vestibulocerebellum or related cerebellar–brainstem pathways, due to distortion or compression from the cyst.

Nonetheless, some patients might initially exhibit symptoms in the latter half of life or remain completely asymptomatic throughout their entire lifespan [15].

### 4.3. Treatment Strategies

DWM with syringomyelia in adults is extremely rare, and there are few guidelines for treatment, which are based on case reports and small series. However, surgery remains the primary treatment for patients with DWM, and commonly reported procedures include posterior fossa decompression, ventriculoperitoneal shunting, and cystoperitoneal shunting [5,20]. Syringoperitoneal shunting has also shown effectiveness [28]. Various surgical techniques have been employed, such as sub-occipital decompression with C1 laminectomy and duroplasty [4,9,11,20] and cyst decompression with obex plugging [8]. Some researchers suggest tailoring the surgical approach based on the degree of aqueductal and subarachnoid space obstruction [29]. Additionally, combined arachnoid adhesiolysis and endoscopic third ventriculostomy (ETV) have also been reported as effective options [15]. Postoperative outcomes were uniformly successful, demonstrating both marked syrinx resolution on neuroimaging and swift clinical recovery in all patients.

## 5. Conclusions

The occurrence of DWM accompanied by syringomyelia in adults is extremely rare. The infrequency of this condition complicates the development of standardized treatment guidelines, highlighting the need for additional research and case studies to enhance our understanding and treatment options. Simple fenestrations of the DW cyst membrane may serve as an effective technique for symptom improvement and syringomyelia resolution.

## Figures and Tables

**Table 1:** Literature review.

CASE	AGE/GENDER	SYRINGOMYELIA	CLINICAL FEATURES	TREATMENT	POSTOPERATIVE OUTCOME	COMMENT
Gardner WJ, et al. [11]	24, F	Cervical	▪ Ataxia ▪ Severe headache ▪ VI nerve palsy ▪ Loss of thermal perception in both arms and hands	Occipitovertebral decompression	▪ Greatly improved, except ataxia ▪ Impaired thermal perception in the left hand and the right arm	The initial suspected diagnosis was Chiari malformation; however, during the surgery DWM was discovered. Exposed cystic appearance of the spinal cord extending below the medulla (communicating syrinx in the form of 5th ventricle)
Baker GS, et al. [8]	25, F	Cervicothoracic	▪ Back pain ▪ Right lower extremity weakness	Cyst decompression and opening of obex	▪ Clinical improvement ▪ at 3-month follow-up	N/A
Hammond CJ, et al. [12]	39, M	Cervicothoracic	▪ Headache ▪ Stabs of pain over the occiput while coughing	CPS revision	▪ Patient’s headaches resolved ▪ Syrinx decrease in size of the at 12-month follow-up	Non-communicating syrinx
Owler BK, et al. [13]	29, F	Cervical	▪ Occipital headache ▪ Upper extremities paresthesia	Suboccipital craniectomy, C1 laminectomy and duroplasty	▪ Transient postoperative dorsal column dysfunction ▪ Complete regression of the syrinx at 6-month follow-up	Non-communicating syrinx
Zhang N, et al. [15]	33, M	Cervicothoracic	▪ Ataxia ▪ Numbness in both hands ▪ Memory deterioration ▪ Urinary incontinence	Two-stage surgery: arachnoid adhesyolisis and ETV	▪ Hands numbness, ataxia and urinary incontinence were greatly improved a few days after surgery ▪ Complete regression of syrinx at 6-month follow-up	Non-communicating syrinx
Wang Y, et al. [14]	19, M	Cervical	▪ Glossal amyotrophy and fibrillation ▪ Weakness of both upper extremities, atrophy of both hands of thenar and hypothenar muscles ▪ Postural tremor on forearms ▪ Absent tendon reflexes of both upper limbs ▪ Brisk tendon reflexes of both lower limbs ▪ Hoffman sign + (left hand) ▪ Romberg sign +	Suboccipital craniectomy, arachnoid adhesiolysis with arachnoid cyst fenestration, spinal cordostomy	▪ Tremor improved ▪ Significant reduction in fourth ventricular size and syringomyelia	Non-communicating syrinx

N/A: not available. +: positive.

## Data Availability

The original contributions presented in this study are included in the article/Supplementary Materials. Further inquiries can be directed to the corresponding author(s).

## Supplementary Materials

The following supporting information can be downloaded at: https://www.mdpi.com/article/10.3390/brainsci15050456/s1, Video S1: Intraoperative video.
